# Visual object tracking challenges revisited: VOT *vs.* OTB

**DOI:** 10.1371/journal.pone.0203188

**Published:** 2018-09-27

**Authors:** Sun Bei, Zuo Zhen, Luo Wusheng, Du Liebo, Lu Qin

**Affiliations:** National University of Defense Technology, Changsha, Hunan, China; University of Tübingen, GERMANY

## Abstract

Numerous benchmark datasets and evaluation toolkits have been designed to facilitate visual object tracking evaluation. However, it is not clear which evaluation protocols are preferred for different tracking objectives. Even worse, different evaluation protocols sometimes yield contradictory conclusions, further hampering reliable evaluation. Therefore, we 1) introduce the new concept of mirror tracking to measure the robustness of a tracker and identify its over-fitting scenarios; 2) measure the robustness of the evaluation ranks produced by different evaluation protocols; and 3) report a detailed analysis of milestone tracking challenges, indicating their application scenarios. Our experiments are based on two state-of-the-art challenges, namely, OTB and VOT, using the same trackers and datasets. Based on the experiments, we conclude that 1) the proposed mirror tracking metrics can identify the over-fitting scenarios of a tracker, 2) the ranks produced by OTB are more robust than those produced by VOT, and 3) the joint ranks produced by OTB and VOT can be used to measure failure recovery.

## 1 Introduction

Object tracking is an essential task in various application scenarios, such as intelligent monitoring, unmanned system operation and human—computer interaction [1]. Numerous tracking approaches have been proposed over the past decades, such as KCF [2], Struck [3], ASLA [4], SCM [5], ECO [6], SiamFC [7], MDNet [8], CCOT [9] etc, have demonstrated superior performance in [10–14]. To measure the performance of these tracking algorithms, much effort has been directed toward building fairly large datasets to facilitate the evaluation process [10–16]. Such studies have focused on building datasets while proposing new methodologies for analyzing tracking performance. However, when researchers have focused on evaluating the performance of trackers, they have often overlooked the reliability and robustness of the evaluation protocols themselves, which could significantly affect the evaluation results.

**Fig 1:**
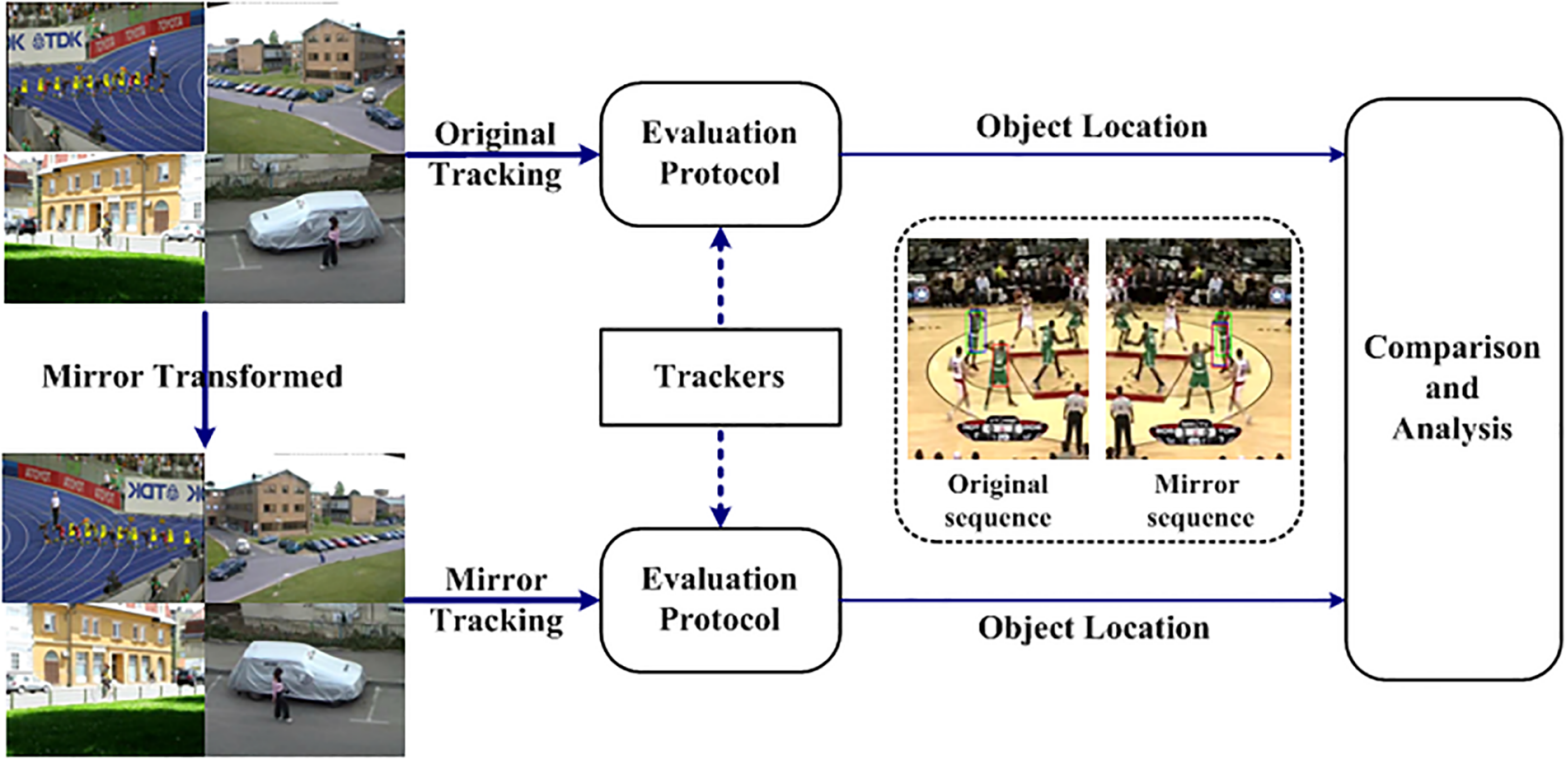
The conducted mirror tracking experiment. In the *basketball* sequence, the green bounding box denotes the ground truth, the blue bounding box indicates the DFT result [18], and the red bounding box indicates the CT result [19]. The performance of CT is dramatically different between the original and mirrored sequences in the mirror tracking experiment, whereas that of DFT remains the same.

A mirrored image represents the same tracking scenario as the original one, thus, trackers should intuitively have the similar performance on a mirrored sequence as on the original one. Moreover, the ranks produced by the same protocol should be consistent. Yang and Patras [17] performed a mirror experiment related to human pose estimation and face alignment, and they found that an object localization model may yield unsymmetrical results on a mirror image, leading to several interesting findings. Therefore, inspired by their work [17], we define the concept of mirror tracking to evaluate the robustness of trackers and evaluation protocols, as shown in Fig 1.

We revisit the milestone tracking challenges OTB [10] and VOT [11] using mirror tracking metrics. We first augmented a publicly available dataset with mirror-transformed versions of the original sequences (32 sequences). The same trackers were run on the same dataset using two evaluation protocols proposed in the two different challenges, i.e., OTB [10] (each tracker is run on each sequence without re-initialization following failure) and VOT [11] (a tracker is re-initialized whenever a failure is detected). We present the results for the original sequences and the mirrored sequences, with the intent of analyzing 1) the trackers’ performance on sequences with different tracking conditions (attributes) and 2) the consistency and robustness of the tracking ranks produced by different evaluation protocols.

Based on our experiments, we can draw the following conclusions: 1) the proposed mirror tracking metrics can help to identify the over-fitting scenarios of a tracker, 2) the rankings produced by the VOT protocol are more sensitive to original vs. mirror tracking than those produced by the OTB protocol, and 3) testing tracker performance using both protocols can yield more accurate information about the tracker’s capability. The contributions of this study are as follows: 1) mirror tracking is introduced to address and analyze the performance of trackers and the robustness of evaluation protocols, and 2) the analysis and conclusions based on mirror tracking for milestone tracking challenges can serve as a reference to advance the study of tracking evaluation tasks.

We review related work in Sec. 2. Details on the dataset construction and evaluation protocols are provided in Sec. 3. Sec. 4 presents and discusses the experimental results. Sec. 5 offers concluding remarks.

## 2 Related work

A tracking algorithm typically consists of three components: target representation, a matching mechanism, and model adaptation [20]. With recent advances in feature representation, both global and local features, such as SIFT, Wavelet, HOF and HOG and CNN features, have been applied to represent objects of interest. Many on-line learning methods have been exploited to achieve sophisticated tracking algorithms with superior performance, e.g., Struck [3] utilizes “Haar features + SVM”, Staple [21] employs fused color and Hog information, several trackers such as SRDCF [22], HCF [23], KCF [2], DCF [24], CFNet [25] and DSST [26] use the popular kernelized correlation filter approaches, while recent state-of-the-art trackers, such as ECO [6], SiamFC [7], MDNet [8, 27] and CCOT [9] are based on CNN features. However, since trackers should not have a large model size, while to obtain an high accuracy, they usually extract a high dimension of features, which can easily lead to a over-fitting problems. Besides, many factors can dramatically affect the tracking performance, such as target deformation, fast motion, illumination conditions, low resolution and occlusion. To improve performance, recent trackers focus on adopting several approaches, including: applying fused CNN features [8], utilizing multi-resolution feature maps [9], reducing computational complexity and providing better diversity of samples [6].

Therefore, it is important to gain a profound understanding of different trackers to advance the state of tracking research. To facilitate tracking evaluation, great efforts have been directed toward the design of benchmark datasets and corresponding evaluation metrics. A significant contribution was made by Wu [10], who collected 50 fully annotated videos and 29 tracking algorithms. This Object Tracking Benchmark (OTB) dataset [28] was later extended with another 50 sequences [29]. In the OTB evaluation protocol, each tracker is provided with an initial bounding box and is run until the end of each video without re-initialization after tracking failure. The authors suggest using the area under the curve (AUC) of either the overlap ratio curve or the center-error distance curve for evaluation. Another milestone work, the Visual Object Tracking (VOT) challenge, was established by Kristan [12] in association with the annual ICCV/ECCV conferences. It is based on two independent metrics: accuracy (the overlap ratio between the tracker and ground-truth bounding boxes) and robustness (measured with respect to the frequency of tracking failure, i.e., when the overlap ratio becomes zero). In the VOT evaluation protocol, whenever a failure occurs, the tracker is re-initialized before it continues tracking. The VOT challenge is held and updated every yearly, nowadays, VOT2016 [13] argued that the averaging ranks of raw accuracy and robustness values ignores the absolute differences, while VOT2017 [14] toolkit performs the OTB no-reset (OPE) evaluations. Both evaluation protocols have attracted considerable attention from the tracking community. However, when researchers have focused on evaluating the performance of trackers, they have often overlooked the reliability and robustness of the evaluation protocols themselves. Therefore, we revisit these two state-of-the-art tracking benchmarks using our proposed *mirror tracking* approach. Rather than focusing on evaluating and ranking the trackers’ performance, we focus on evaluating the tracking challenges themselves using both original and mirrored sequences to see how the performance changes under the same tracking conditions (attributes), thereby gaining a profound understanding of tracking benchmarks that could guide future work on the design of evaluation protocols.

## 3 Dataset and evaluation protocols

### 3.1 Mirror tracking dataset

Note that merely constructing a very large dataset does not guarantee diversity in its visual attributes but significantly slows the evaluation process [12]. A better approach is to perform sequence clustering to reduce the size of the dataset while maintaining its diversity. Based on this approach, the OTB group [10] has developed a challenging and representative dataset consisting of sequences chosen from a large pool by clustering the visual features of the objects and backgrounds. Therefore, we utilized the publicly available TB-50 dataset [10] (including 50 different target objects) to conduct the experiments reported in this paper.

Each test image was flipped horizontally to generate the mirror image. Thus, the entire dataset was doubled in size compared with the original one. To maintain consistency of the ground truths, the coordinates were adapted accordingly. We denote an original sequence by *P* = {*I*_*k*_}. The corresponding mirror sequence is denoted by $\hat{P}=\{{\hat{I}}_{k}\}$, where *k* = {1, 2, 3, …, *n*}, ${\hat{I}}_{k}$ is the mirror image of *I*_*k*_, and *n* is number of frames in the sequence. In the original sequence, the object is represented by a bounding box defined by four variables, denoted by {*x*, *y*, *w*, *h*}, where {*x*, *y*} is the top left corner and {*w*, *h*} represents the corresponding width and length. Accordingly, the coordinates of the object in the mirror image are {*W*_*I*_ − (*x* + *w*), *y*, *w*, *h*}, where *W*_*I*_ is the width of the image. Since the tracking conditions are not changed in the mirror images, the associated attributes of each image are the same as those of the original one.

### 3.2 Evaluation protocols

Two different tracking evaluation protocols have attracted considerable attention, namely, the OTB [10] and VOT [12] protocols. The main difference between VOT and OTB is that in OTB, each tracker is run on each sequence without re-initialization following failure, whereas in VOT, a tracker is re-initialized whenever a failure is detected.

#### OTB evaluation protocol

The OTB protocol was proposed by Wu et al. [10] in CVPR2013 and defines two means of evaluating tracking robustness: temporal robustness evaluation (TRE) and spatial robustness evaluation (SRE). TRE and SRE represent an improvement over the conventional one-pass evaluation method (OPE), in which each tracker is initialized only on the first frame; in TRE, the tracker is initialized on a different frame (i.e., with a temporal spread), and in SRE, the tracker is initialized with a noisy bounding box (i.e., with a spatial spread). Furthermore, the OPE, TRE and SRE approaches all consider both precision and accuracy to evaluate the tracking performance.

The accuracy in frame *k* is defined as the bounding box overlap *φ*_*k*_, which is calculated using the tracker-output bounding box *TT*_*K*_ and the ground-truth bounding box *GT*_*k*_ as shown in Eq 1:

$$\varphi_{k}=\frac{|TT_{k}\cap GT_{k}|}{|TT_{k}\cup GT_{k}|}$$ (1)

where ∩ and ∪ represent the intersection and union of two regions, respectively, and |•| is the region size measured as the number of pixels.

The precision is quantified by the center location error, which measures the difference between the center location $X_{k}^{T}$ predicted by the tracker and the ground-truth center location $X_{k}^{G}$ in the *k*th frame. It is often defined as a root-mean-square error (RMSE):

$$RMSE=\sqrt{\frac{1}{N}\sum_{k=1}^{N}∥X_{k}^{G}-X_{k}^{T}∥}$$ (2)

For a range of values of the accuracy ratio *φ*_*k*_, a success curve is drawn. The final score is calculated as the AUC to represent the overall tracking performance.

#### VOT evaluation protocol

The VOT challenge is organized every year by Kristan et al. [12] in association with ICCV/ECCV. Since the center location error is sensitive to subjective human-selected bounding boxes, the VOT protocol uses only the overlap to define both robustness and accuracy. The accuracy defined in VOT is the same as that defined in OTB. For robustness, the protocol specifies an overlap threshold to determine tracking success. The number of correctly tracked frames is then divided by the total number of frames, as shown in Eq 3, to achieve a more suitable evaluation:

$$P_{\tau }\left(GT,TT\right)=\frac{\left|\right|{\left\{t|\varphi_{k}>\tau \right\}}_{k=1}^{N}\left|\right|}{N}$$ (3)

where *τ* is the overlap threshold and *N* is the run time of the tracker in frames. A failure is identified in a frame when the overlap (as computed using Eq 1) is below the defined threshold *τ* (zero in the present experiments). The normalized number of correctly tracked frames is used to represent the robustness of the tracker.

## 4 Experiments and results

We selected ten trackers in the experiments by VOT and OTB, namely, CT [19], CSK [30], ORIA [31], DFT [18], IVT [32], ECO [6], MDNet [8], CCOT [9], Staple [21] and KCF [2] as summarized in Table 1. Each tracker represents a different combination of the target representation, search mechanism and matching method. In the experiments, we implemented the trackers with the same parameters on the same dataset using different evaluation protocols. This was done to conduct a fair comparison between the OTB and VOT tracking challenges. The experiments were designed to 1) identify the scenarios in which the trackers exhibit over-fitting problems; 2) analyze the robustness evaluation of different protocols, i.e., OTB [10] and VOT [12]; and 3) make detailed discussion of mirror tracking with trackers and protocols.

**Table 1:** The trackers tested in the experiments. HT: holistic template; LT: local template; DF: distribution fields; DM: discriminative model; GM: generative model.

Method	Representation	Search mechanism	Matching method
CT [19]	HT, Haar, DM	Dense sampling	Naive Bayes classifier
CSK [30]	HT, DM	Dense sampling	Max Response
ORIA [31]	HT, GM	Local optimum	Sparse representation
DFT [18]	LT, DF	Local optimum	*L*_1_ distance
IVT [32]	HT, PCA, GM	Particle filter	Euclidean distance
ECO [6]	CNN	Correlation filter	*L*_2_ Norm distance
MDNet [8]	CNN	long-term,short-term	Bounding box regression
CCOT [9]	CNN	Correlation filter	Max Confidence
Staple [21]	Hog, Color	Correlation filter	Response map
KCF [2]	Gray scale, Hog	Correlation filter	Kernel regression

### 4.1 Tracking results using the OTB protocol

**Fig 2:**
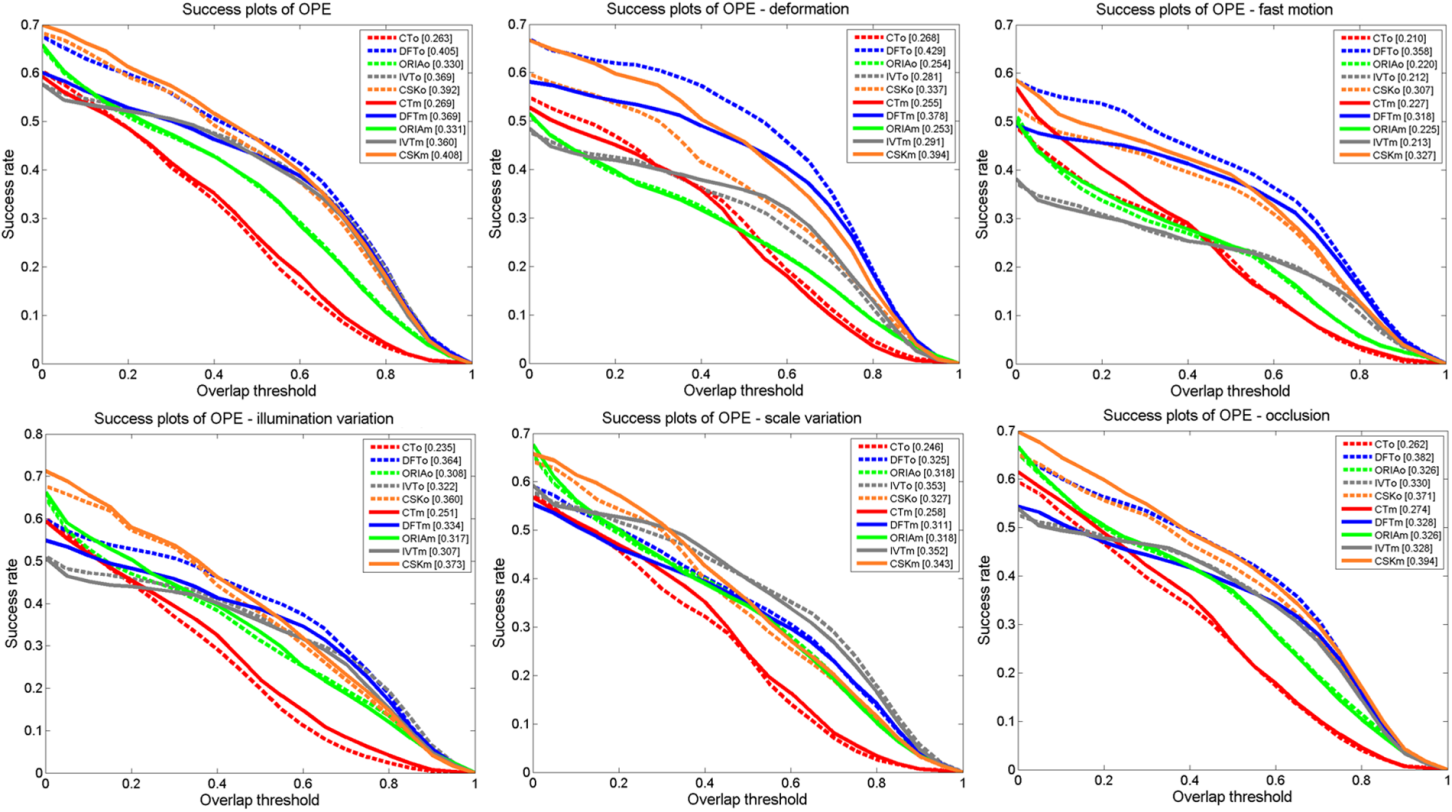
Success plots based on the OTB evaluation protocol. A tracker name with a subscript “o” indicates that the tracker was tested on the original sequences, whereas a subscript “m” denotes mirror tracking.

The tracking results on the entire dataset and on sub-datasets with corresponding attributes are presented in Fig 2. In this section, we mainly report the OPE results based on the OTB protocol. Curves of the same color represent tracking results from the same tracker, and the AUC scores are also presented in Fig 2. A solid line denotes mirror tracking, whereas a dashed line represents original tracking.

With the OTB evaluation protocol, we find that the overall performance of each tracker is quite similar between mirror tracking and original tracking. Since the original and mirrored sequences share the same attributes (represent the same scenarios), one expects consistent performance on both tracking sets. However, we can see significant difference for 1) DFT in scenes with target deformation, fast motion, and occlusion; 2) CT in scenes with fast target motion, deformation and occlusion; and 3) IVT in scenes with variations in target illumination. By contrast, CSK and ORIA achieve consistent performance between original and mirror tracking. We conclude that because the different trackers exhibit different variations in performance between original and mirror tracking, these differences may be related to the different realization principles of the trackers. Furthermore, it is clear that since both the original and mirrored sequences represent the same tracking scenarios, the observed tracking differences indicate over-fitting problems encountered by the tested trackers under particular tracking conditions, such as illumination variations, scale variations, and motion blur.

Note that even though a tracker might perform differently on the original and mirrored sequences, the ranks produced by OTB within each tracking set (original tracking and mirror tracking) are very consistent. Moreover, the trackers that are highly ranked on the original sequences also achieve the same high ranks in mirror tracking, which indicates that the OTB evaluation protocol produces a very robust ranking.

### 4.2 Tracking results using the VOT protocol

**Fig 3:**
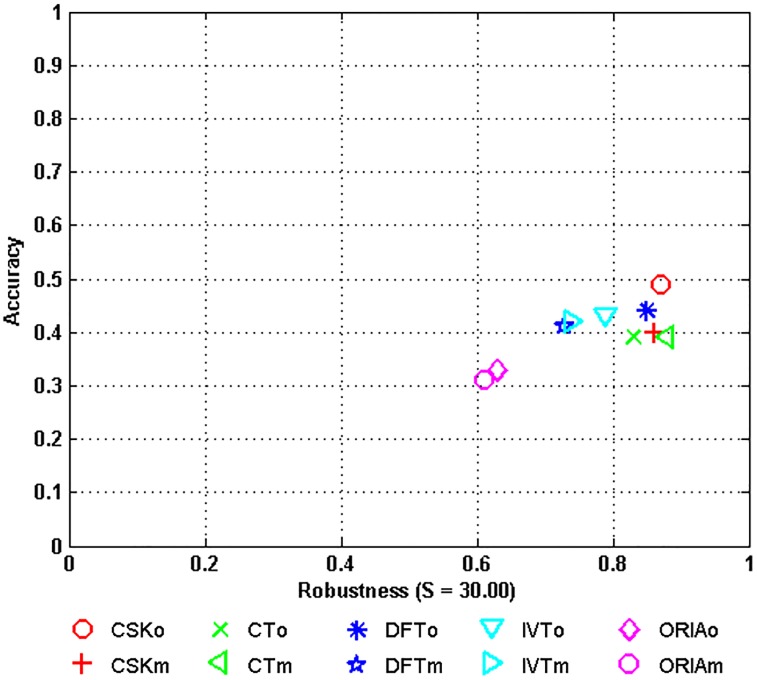
AR plots of original and mirror tracking. The results of the same tracker on both the original and mirrored sequences are marked with the same color.

The tracking results of each tracker when tested on the entire dataset using the VOT evaluation toolbox are presented in Fig 3 and Table 2. In Fig 3, the closer a tracker lies to the upper right corner, the better its performance is.

**Table 2:** Tracking performances evaluated using the VOT evaluation protocol. The overall ranking score is produced by the VOT toolbox as described in [12].

Raw results	CSKo	CTo	DFTo	IVTo	ORIAo	CSKm	CTm	DFTm	IVTm	ORIAm
Overlap	0.49	0.39	0.44	0.43	0.33	0.40	0.39	0.41	0.42	0.31
Failures	3.61	4.22	4.09	4.33	6.88	3.91	3.58	4.43	4.41	6.92
Overall rank	2.29	2.78	2.35	2.78	4.78	2.86	2.47	3.03	3.20	3.44

In Table 2, differences in performance between original tracking and mirror tracking can be seen for IVT (w.r.t. overlap), CSK (w.r.t. overlap), DFT (w.r.t. failures) and CT (w.r.t. failures). These findings show that these four trackers have more severe over-fitting problems compared with ORIA. To facilitate a deeper understanding of the results, we also present accuracy and robustness plots (AR plots) of the tracking results on the entire dataset in Fig 3. From the AR plots, we find results similar to those obtained for the OTB evaluation protocol: tracking differences are again found for CT, DFT and IVT, whereas ORIA achieves consistent performance in original and mirror tracking. However, unlike in the case of the OTB protocol, in VOT, the ranking of the same tracker varies dramatically between the original and mirrored sequences; e.g., in Table 2, IVT ranks third in original tracking but holds first place in mirror tracking, whereas CSK holds first place in original tracking but ranks third in mirror tracking.

We remark that 1) a tracker with a larger performance difference between original tracking and mirror tracking has more severe over-fitting problems under those particular tracking conditions (associated with the corresponding attributes) and 2) the rankings produced by the VOT protocol are more sensitive to original vs. mirror tracking than those produced by the OTB protocol.

### 4.3 OTB protocol *vs.* VOT protocol

We present the different rankings of the tested trackers in Table 3. Four different rankings were produced using each evaluation protocol, i.e., the ranks on the original sequences, the ranks on the mirrored sequences, the average ranks on both the original and mirrored sequences, and the overall ranks considering both sets of sequences. We can see that the ranks produced by the OTB protocol are highly consistent (the top-ranked trackers on the original sequences also earn the top ranks for mirror tracking, with the exceptions of CSK and DFT), whereas the VOT protocol produces less robust rankings. One possible reason is that the trackers might encounter failure in different frames between the original and mirrored sequences, and in the VOT protocol, a tracker is re-initialized after each failure, which could significantly alter the conditions and status of the tracking process.

**Table 3:** The ranks of the tested trackers based on the OTB and VOT protocols. In OTB, the trackers are ranked based on their AUC scores, whereas VOT provides an overall ranking score that combines robustness and accuracy. A subscript “o” indicates that the tracker was tested on the original sequences, whereas a subscript “m” indicates that the tracker was tested on the mirrored sequences. A tracker name without any subscript indicates the results for the entire dataset.

Ranking	OTB				VOT			
	Original	Mirrored	Average rank	Overall rank	Original	Mirrored	Average rank	Overall rank
1	DFTo (0.405)	CSKm (0.408)	CSK (0.400)	CSKm	CSKo (2.29)	CTm (2.47)	CSK (2.58)	CSKo
				DFTo				DFTo
2	CSKo (0.392)	DFTm (0.369)	DFT (0.387)	CSKo	DFTo (2.35)	CSKm (2.86)	CT (2.63)	CTm
				DFTm				CTo
3	IVTo (0.369)	IVTm (0.360)	IVT (0.364)	IVTo	CTo (2.78)	DFTm (3.03)	DFT (2.69)	IVTo
				IVTm				CSKm
4	ORIAo (0.330)	ORIAm (0.331)	ORIA (0.330)	ORIAm	IVTo (2.78)	IVTm (3.20)	IVT (2.99)	DFTm
				ORIAo				IVTm
5	CTo (0.263)	CTm (0.269)	CT (0.266)	CTm	ORIAo (4.78)	ORIAm (3.44)	ORIA (4.11)	ORIAm
				CTo				ORIAo

Furthermore, the average ranks of the trackers also generally differ between the two evaluation protocols, as seen from Table 3. This difference arises from the different evaluation objectives. OTB performs temporal and spatial robustness evaluations by specifying different initial frames and utilizing different initial boxes obtained by shifting or scaling the ground truth, whereas VOT performs re-initialization after five frames of tracking failure. OTB is more suitable than VOT for testing trackers that are good at automatically recovering from failures because if a tracker misses the target only in the temporal dimension, the re-initialization in the VOT protocol can dramatically affect the final ranking, whereas the OTB protocol is more robust to such short-term failures, moreover, OTB protocol is more similar to real tracking conditions due to the lack of re-initialization after failure. However, the OTB protocol also has some limitations: when a tracker fails early in a sequence, the OTB protocol will show a low success ratio, whereas if it fails late in a sequence, a high success ratio may be reported. This problem may be mitigated by the VOT protocol, which re-initializes the tracker and counts the number of failures to measure the tracking robustness. Moreover, OTB is never updated since publication, and consequently, trackers can achieve good results through extensive parameter tuning, whereas the VOT challenge is held yearly and updated every time, which is beneficial for obtaining realistic tracker ranks and helping to improve tracker performance, such as, VOT2015 [12] toolkit proposed more carefully annotated sequences (60) and better evaluation indicators (e.g., EAO), and VOT2017 [14] toolkit also performs the OTB no-reset (OPE) experiment. Thus, more informative conclusions might be obtained by testing a tracker using both protocols. In this way, we can comprehensively evaluate the trackers’ performance under both the VOT protocol and the OTB protocol, as summarized in Table 4.

**Table 4:** Evaluation conclusions from both the OTB and VOT protocols.

	VOT	OTB	Evaluation conclusion
Tracking performance	Good	Good	Well-performing tracker
	Good	Poor	Automatic recovery from failure
	Poor	Good	Failure early in the sequence
	Poor	Poor	Poorly performing tracker

### 4.4 Mirror tracking and trackers

This paper provides a simple concept of mirror tracking and further explores tracking evaluation using two state-of-the-art challenges, VOT and OTB. From the proposed mirror tracking, we find the over-fitting problems, actually, we consider this is due to the basic structure of trackers. To prove this, we further select five more state-of-the-art trackers for mirror tracking test. Among them, MDNet, ECO and CCOT utilize CNN based features, staple extracts the fused color histogram and Hog information, while KCF employs the popular kernelized correlation filter. Table 5 illustrates the evaluation results (accuracy and robustness) based on VOT2017 [14], including mirror tracking and original tracking. From the results, we find: 1) mirror errors still exist in the state-of-the-art trackers, 2) the ranking of state-of-the-art trackers is more robust than the previous trackers in the mirror and original tracking, and 3) trackers have stronger abilities in dealing with over-fitting problems can result in a less mirror errors.

**Table 5:** Tracking performances based on the VOT2017 [14] protocol. The results are produced by the VOT toolbox as described in [12]. The index with “o” denotes original tracking, while “m” presents the results of mirror tracking.

Raw results	ECO	CCTO	MDNet	Staple	KCF	DFT	CSK	IVT	ORIA	CT
Accuracy (o)	0.483	0.494	0.511	0.530	0.447	0.413	0.432	0.400	0.365	0.374
Accuracy (m)	0.482	0.490	0.508	0.524	0.435	0.395	0.388	0.420	0.351	0.358
Robustness (o)	0.276	0.318	0.698	0.688	0.773	1.521	1.408	1.639	2.512	1.614
Robustness (m)	0.281	0.325	0.689	0.692	0.786	1.536	1.458	1.654	2.585	1.718

**Fig 4:**
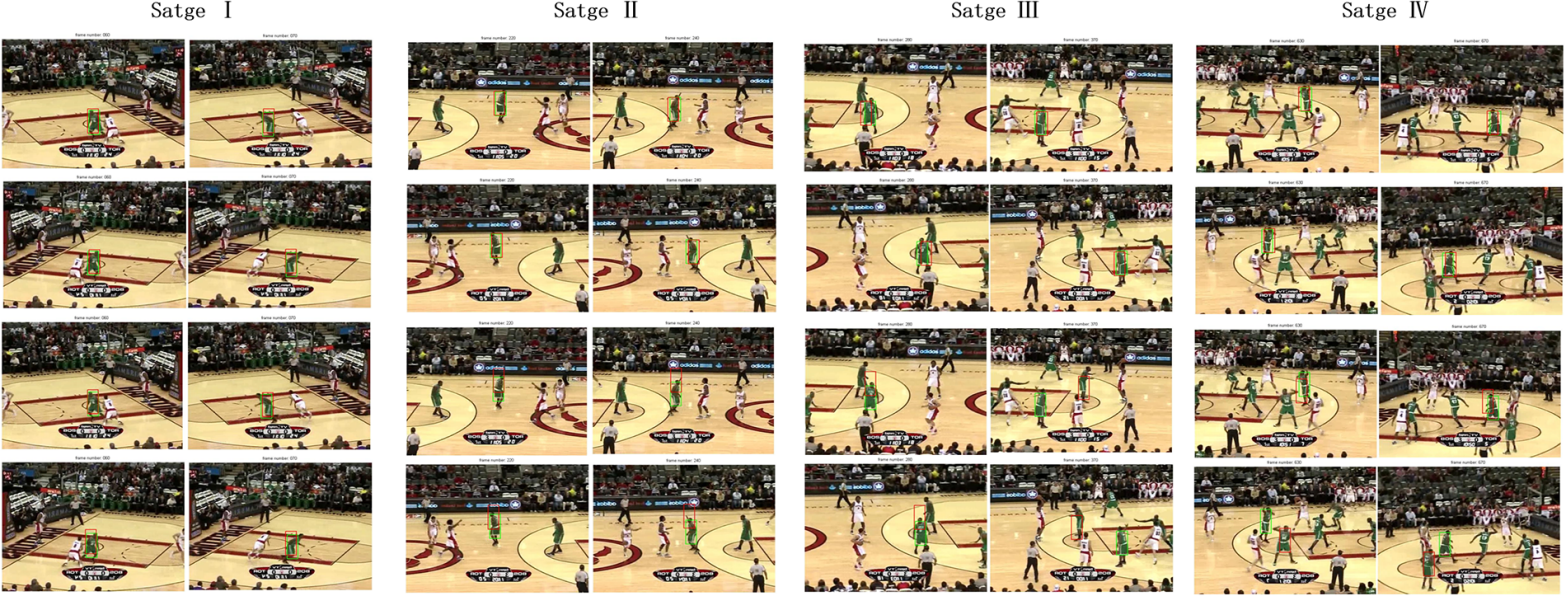
The tracking results of ECO and CT on the original and mirror “Basketball” sequences. Where the first two rows respectively denote the results of ECO in original tracking and mirror tracking, and the next two rows represents the results of CT. The blue box is ground truth, and the red box is the output of tracker.

We select ECO and CT for further comparisons, in which CT relies the typical ‘features+machine learning’ mechanism, while ECO is based on the CNN features. We illustrate their tracking results in original and mirror “basketball” sequences in Fig 4. As shown in Fig 4, we divide the sequences into four stages for analysis, ECO is very stable in the overall process of original and mirror tracking, yet CT performs worse, such as in stage one, it can basically accurate locate the target, while in stage two, it begins to lose the target, and then in stage three completely lose it. Moreover, during the first three stages, CT performs nearly the same in both original tracking and mirror tracking, but in stage four, we surprisingly find CT re-locate the target in original tracking but still lose it in mirror tracking. One possible reason is that ECO based on CNN should perform better than CT using traditional machine learning. Moreover, ECO reduces the model parameters and provides better diversity of samples. Considering mirror and original sequences describe the same scenes but lead to different tracking results, we consider mirror error comes from over-fitting problems of trackers itself. Actually, since trackers should not adopt a large model size, while to improve accuracy, they usually extract high-dimension of features, and this can easily results in over-fitting problems. More seriously, when a tracker locates a wrong target position, it will generate different dataset samples and then result in different model parameters, which would affect the tracking performance in the next frames.

Based on this, the improvements of over-fitting problems include: 1) conducting more types of samples in model updating, e.g., by rotation and mirror-transformed, and 2) utilizing fused features, e.g. CNN features, Gray, Color information, Hog etc, and 3) reduces the number of parameters.

### 4.5 Mirror tracking and evaluation protocols

Existing evaluation protocols mainly adopt center error and average overlap rate, while the center errors mainly focus on target center location and cannot measure deformation, and the average overlap rate results can be influenced by different selected thresholds and metrics. However, two trackers obtain a same center errors may output completely different locations. Considering the target position will influence the selection of training samples, and then result in model updating differences, trackers will output different performance in original and mirror tracking, such as in Fig 4. Based on the proposed mirror tracking, we: 1) provide an extension of the available sequences with same attribution, the existed annotation of sequences is based on experience and in manually, while mirror sequences provide exactly the same attributions as original sequences; 2) help identify the over-fitting problems and yield more robust evaluation, since mirror error comes from over-fitting problems, while mirror sequences provide exactly the same attributes as original sequences, so it can be used for robustness evaluation of trackers; 3) combined using original and mirror tracking can help locate frames where over-fitting occurs and then contribute to tracker improvement works, since the frame with large mirror errors probably denotes the occurrence of over-fitting problems, based on this, researchers can analyze why over-fitting problems occurs in such frames, and conduct improvements, e.g., selecting more diversity of training samples, such as by rotation and mirror-transformed, or optimizing parameter models.

Moreover, the mirror tracking does not conflict with the existing protocols. It only requires to make a mirror-transformed of sequences, and then conduct evaluation on both original and mirror sequences using the existing protocols. Performing mirror tracking in combination with existing protocols and comparing the original and mirror tracking results have the following advantages: 1) provide an extension of the available testing sequences, 2) identify the over-fitting problems and yield more robust evaluation, and 3) locate frames which over-fitting occurs and help improving the selection of training set.

## 5 Conclusion

In this paper, we have proposed a novel mirror tracking methodology to evaluate the performance of state-of-the-art trackers and have also revisited state-of-the-art tracking evaluation protocols using the same trackers tested on the same dataset. It is concluded that: 1) the over-fitting problems of trackers is really existing, and trackers that exhibit larger differences in original and mirror tracking performance are subject to performs worse under those particular tracking conditions, e.g. deformation, fast motion, and occlusion, 2) the rankings produced by the VOT protocol are more sensitive to original and mirror tracking than those produced by the OTB protocol, and 3) combined using mirror tacking with original tracking can contribute providing more accurate evaluation about the tracker’s capability. The conclusions drawn from this paper could lead to future advances in evaluation protocol construction.
